# Lymphocyte Autophagy in Homeostasis, Activation, and Inflammatory Diseases

**DOI:** 10.3389/fimmu.2018.01801

**Published:** 2018-08-06

**Authors:** Florent Arbogast, Frédéric Gros

**Affiliations:** ^1^CNRS UPR3572, Immunology, Immunopathology and Therapeutic Chemistry/Laboratory of Excellence MEDALIS, Institut de Biologie Moléculaire et Cellulaire, Strasbourg, France; ^2^University of Strasbourg, Strasbourg, France

**Keywords:** autophagy, mitophagy, metabolism, unfolded protein response, autoimmunity, lymphocytes

## Abstract

Autophagy is a catabolic mechanism, allowing the degradation of cytoplasmic content *via* lysosomal activity. Several forms of autophagy are described in mammals. Macroautophagy leads to integration of cytoplasmic portions into vesicles named autophagosomes that ultimately fuse with lysosomes. Chaperone-mediated autophagy is in contrast the direct translocation of protein in lysosomes. Macroautophagy is central to lymphocyte homeostasis. Although its role is controversial in lymphocyte development and in naive cell survival, it seems particularly involved in the maintenance of certain lymphocyte subtypes. Its importance in memory B and T cells biology has recently emerged. Moreover, some effector cells like plasma cells rely on autophagy for survival. Autophagy is central to glucose and lipid metabolism, and to the maintenance of organelles like mitochondria and endoplasmic reticulum. In addition macroautophagy, or individual components of its machinery, are also actors in antigen presentation by B cells, a crucial step to receive help from T cells, this crosstalk favoring their final differentiation into memory or plasma cells. Autophagy is deregulated in several autoimmune or autoinflammatory diseases like systemic lupus erythematosus, rheumatoid arthritis, multiple sclerosis, and Crohn’s disease. Some treatments used in these pathologies impact autophagic activity, even if the causal link between autophagy regulation and the efficiency of the treatments has not yet been clearly established. In this review, we will first discuss the mechanisms linking autophagy to lymphocyte subtype survival and the signaling pathways involved. Finally, potential impacts of autophagy modulation in lymphocytes on the course of these diseases will be approached.

## Introduction

Autophagy is a catabolic process related to lysosomal activity. Several forms of autophagy coexist in vertebrate cells. They have been historically separated into three major mechanisms: macroautophagy, chaperone-mediated autophagy (CMA), and microautophagy. But in fact, as mentioned later, in each form, several mechanism subtypes have been described, with different physiological roles and particular molecular effectors. Microautophagy, consists in the direct invagination of lysosomal membrane, to engulf cytoplasmic content for degradation. The physiological relevance of this autophagy pathway is not studied in lymphocytes. We will thus focus on macroautophagy and CMA in this review.

Macroautophagy occurs through the formation of double-membrane vesicles named autophagosomes, which emerge from structures named called phagophores. It has been described that autophagosome formation can occur at the contact site between endoplasmic reticulum (ER) and mitochondria, in structures named omegasomes. Some studies also propose the plasma membrane as a source for autophagy initiation in certain contexts such as primary cilium formation (1). Macroautophagy execution is under the control of autophagy-related (*ATG*) gene products, conserved from yeast to vertebrates and extensively reviewed elsewhere (2). CMA is a selective form of autophagy allowing the direct translocation of protein substrates from the cytosol into the lysosomal lumen. Although cross-regulations with macroautophagy have been described, CMA does not rely on ATG activity but on the expression of the chaperone HSPA8/Hsc70 and lysosomal-associated membrane protein 2 isoform a (LAMP2a).

## The Macroautophagy Machinery

**Figure 1:**
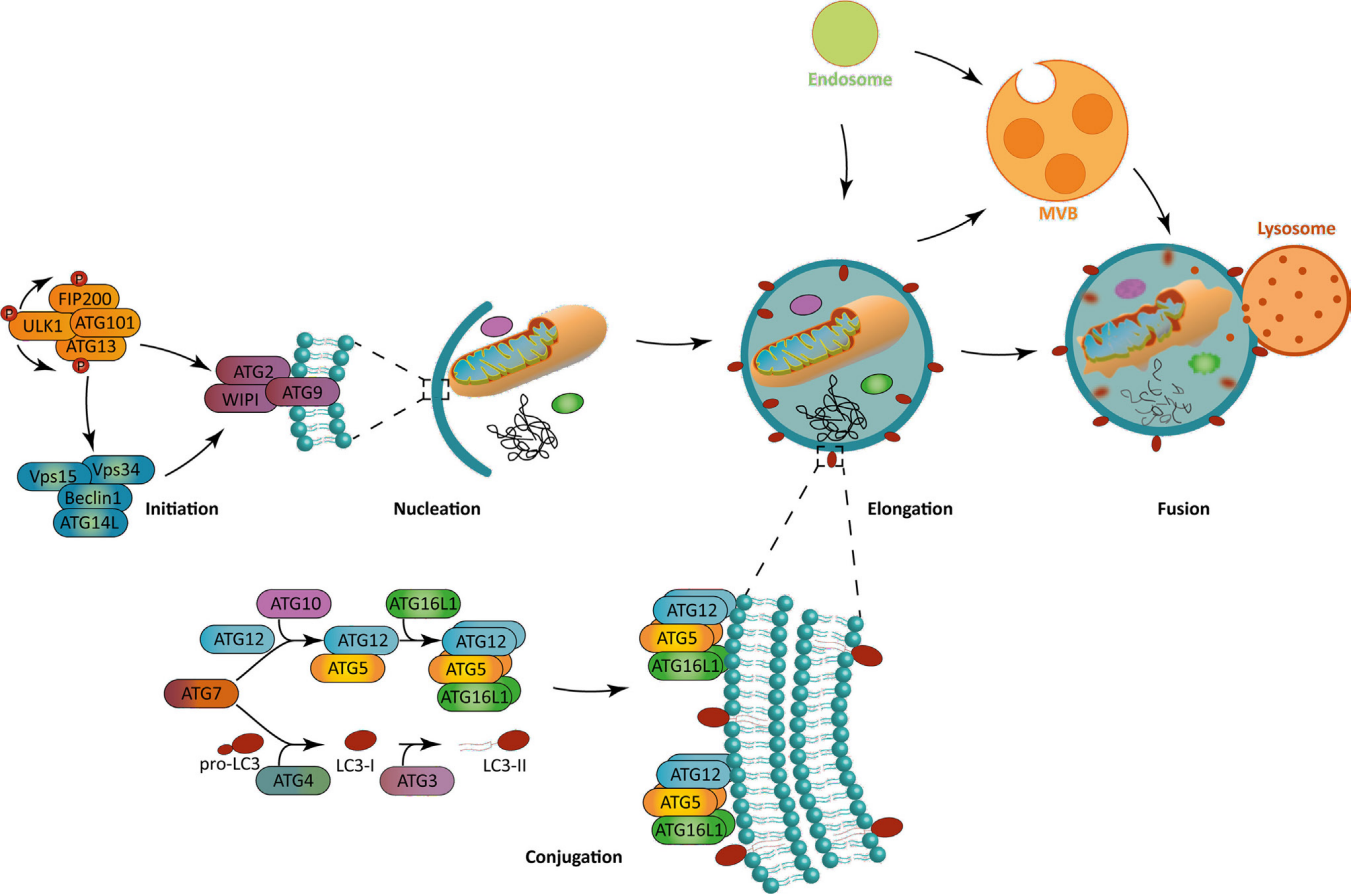
The macroautophagy process. (Left) Autophagy initiation is mediated in a context-dependent manner by ULK1 complex, Beclin-1 complex, or both. These complexes allow the recruitment to the phagophore assembly site of the further effectors ATG8, WIPI, and ATG2 during the nucleation step. (Bottom) The ATG12-ATG5/ATG16L1 complex allows the incorporation of LC3-II in the phagophore, which is crucial for its elongation. Both ATG12-ATG5/ATG16L1 complex and LC3-II are formed by the combined action of two ubiquitin-like systems. While the first one mediates ATG5 complex formation, the second one is responsible for the pro-LC3 cleavage to form LC3-I and a further addition of a phosphatidylethanolamine residue on it to form LC3-II. (Right) Macroautophagy allows the engulfment of cytoplasmic portions. The fusion with lysosomes leads to the degradation of autophagosomal content. Alternatively, autophagosomes can fuse with endocytic vesicles or multivesicular bodies, prior to fusion with lysosomes. The blue lipids layers represent the phagophore membrane. Abbreviations: ATGs, autophagy-related genes; FIP200, FAK-family interacting protein of 200 kDa; LC3, short for MAP1LC3 microtubule-associated protein 1 light chain 3; ULK1, Unc-51 like autophagy activating kinase 1; Vps15/34, vacuole protein sorting 15/34; WIPI, WD-repeat *protein* interacting with phosphoinositides.

Two main activating pathways control the phagophore formation (Figure 1). The first one is the mammalian target of rapamycin (mTOR)-ATG1/unc-51 like autophagy activating kinase (ULK1) axis, which regulation is tightly linked to the metabolic state of the cell. Indeed, macroautophagy was first described as a response to nutrient depravations. In these conditions, for example, amino acid starvation, mTOR pathway is inhibited which leads to ULK1 kinase activation and autophagy initiation. Alternatively, the adenosine monophosphate-activated protein kinase (AMPK) can be activated under nutrient stress, leading both to ULK1 activation and mTOR inhibition, ultimately inducing autophagy. The second, and major, pathway involves the generation of phosphatidylinsositol-3-phosphate (PInst3p) by Beclin-1-BECN1/Vps34 complex, exerting a class III phosphatidylinositol 3 kinase (PIK3CIII) activity. ULK1 complex can directly phosphorylate and activate Beclin 1/Vps34 complex after its recruitment to the initiation site, but also indirectly trigger it *via* AMBRA1 phosphorylation. Depending on the context, only ULK1, Beclin 1/Vps34 pathway, or both are necessary for autophagy initiation. Non-canonical forms of autophagy have indeed been described, needing only parts of core ATGs for initiation or for further steps (3). The formation of the phagophore can give rise to the autophagosome at the elongation phase. During this step, the ATG7 and ATG10 ubiquitin-ligase-like (E1 and E2-like, respectively) allow the covalent conjugation between ATG5 and ATG12, which can then recruit ATG16L1. PInst3P generated by Beclin1/Vps34 complex activity allows the recruitment of molecules like members of the WD-repeat protein interacting with phosphoinositides (WIPI) family that indicate the site of elongation by recruiting ATG12-ATG5/ATG16L1 complex. The latter leads to the conjugation of microtubule-associated protein light chain 3 (MAP1LC3), often abbreviated LC3, with a phosphatidylethanolamine (PE) that can be integrated into the autophagosomal membrane. This lipidated form is then named LC3-II, in opposition to LC3-I referring to the soluble cytosolic form. Other members of LC3 family, such as GAPARAP (gamma-aminobutyric acid A receptor) proteins can also associate with autophagosome membranes. Before lipidation, LC3 is processed by ATG4 to expose a glycine at the C-terminal domain. The E1-like ligase ATG7 activates LC3 C-terminal glycine residue forming with it a thioester bond. The E2-like ligase ATG3 then replaces ATG7 allowing the action of ATG5-ATG12/ATG16L1 as a putative E3-like enzyme, transferring PE to LC3. ATG5-ATG12/ATG16L1 complex is present on the autophagosomal membrane until vesicle closure, whereas LC3-II remains associated during the whole autophagic process. The closed autophagic vesicle is then addressed to lysosomes during the maturation phase. The low pH and the activity of degradative enzymes lead to the digestion of the autophagosome content in a so-called autolysosome. Macroautophagy was first thought to be largely non-specific, regarding the nature of the cytoplasmic content targeted for degradation. It is now clear that several forms of macroautophagy coexist, selecting organelles, protein aggregates, microorganisms, for degradation (4). This selectivity is ensured by cargo-specific adapter proteins that contain LC3 interacting regions (LIR), which can dock to LC3 expressed on autophagosomes, ultimately leading them to degradation.

## Autophagy, Glucose, and Lipid Metabolism

Although the role of autophagy during amino acid starvation has been extensively studied, it appears that autophagy is also modulated by glucose availability and involved in lipid metabolism. Indeed, mTOR complex 1 (mTORC1) is not only activated during amino acid starvation but also under limited glucose availability, independently of AMPK activity (5). It has been described that inhibition of hexokinase II (HK2), enzyme essential for glycolysis, by 2-deoxyglucose leads to inhibition of autophagy. In cardiomyocytes, HK2 specifically induces autophagy in the absence of glucose, protecting cells from death. HK2 can directly bind mTORC1 complex, inhibiting its activity, and thus inducing autophagy. In the presence of glucose-6-phosphate generated by HK2 activity under glucose availability, mTOR binding to HK2 is abolished and autophagy is inhibited.

Autophagy also plays a role in free fatty acid (FFA) mobilization. Lipid droplets (LDs) are organelles present in every eukaryotic cell. In specialized tissues, like adipose tissue and liver, LDs are needed for energy storage. During lipolysis, a multi-protein complex mainly assures FFA mobilization from LDs (6). However, in cells expressing few lipolysis enzymes, during starvation or increased energy demand, macroautophagy is required to execute LD degradation and a quick increase in FFA availability (7, 8). This selective form of macroautophagy was named lipophagy, and while it consists in a ubiquitous pathway, yet it remains poorly characterized. However, as for other forms of selective autophagic degradation, several receptors, such as huntingtin and p62, might be recruited to facilitate LD degradation (9, 10). Increase in FFA availability is crucial to generate succinate, the primary substrate for mitochondrial β-oxidation, but also for gluconeogenesis thus creating fuel for glycolysis and so increasing adenosine tri-phosphate (ATP) level (11). However, increasing FFA availability might induce cellular toxicity. Consequently, after their mobilization to sustain the energetic demand, some ATGs of the core machinery might induce LD *de novo* formation (12). LD generation is also needed to limit an accumulation of potential toxic lipids such as triacylglycerols, diacylglycerols, and ceramids. LDs are then created to sequestrate those overabundant lipids maintaining cellular homeostasis (13). Therefore, macroautophagy and its machinery play a role in lipid homeostasis by lowering their toxicity potential, as well as in their metabolism by insuring FFA efficient mobilization.

## Selective Forms of Autophagy

Basal macroautophagic activity plays a major role in cell homeostasis. It allows the degradation of malfunctioning organelles, especially mitochondria, part of the ER, lysosomes, and peroxisomes (14). A specialized form of macroautophagy, aggrephagy, can also target ubiquitin-tagged aggregates, which cannot be processed by the proteasome, after ubiquitin linking with p62/SQSTM1 (sequestosome 1) (15). Housekeeping functions of macroautophagy linked to organelle and protein homeostasis are particularly required in cell populations that rely on self-renewal to maintain tissue integrity. For example, long-lived cells like neurons are highly dependent on aggrephagy and mitophagy for proper survival (16). As mentioned above, nutrient stress can induce macroautophagy, leading to the degradation of macromolecules, supporting energy production, and providing new building blocks for synthesis of new molecules.

### Mitophagy

In eukaryotic organisms, mitochondria form a sophisticate and dynamic network, with a central role in energetic production mainly through oxidative phosphorylation (OXPHOS), in relation with glycolysis and/or fatty acid oxidation (FAO). During OXPHOS, electrons are transported in the inner chain of the mitochondria to generate an H^+^ gradient, crucial for the final step of ATP generation. Electron leakage or damaged transport chain might lead to the formation of reactive oxygen species (ROS), which are neutralized by ROS scavenging enzymes. Eventually some ROS might not be neutralized and act on cellular homeostasis. At low levels, ROS have been demonstrated to contribute to cell proliferation and survival. However, at higher levels, their actions as proteins/lipids oxidizers and DNA damages inducers, contribute to tumorigenesis and/or apoptosis. Since accumulation of defective mitochondria directly impairs energetic production and cellular homeostasis, cells might try to repair them *via* fusion/fission mechanisms or degrade them through a selective autophagic pathway named mitophagy (17, 18).

**Figure 2:**
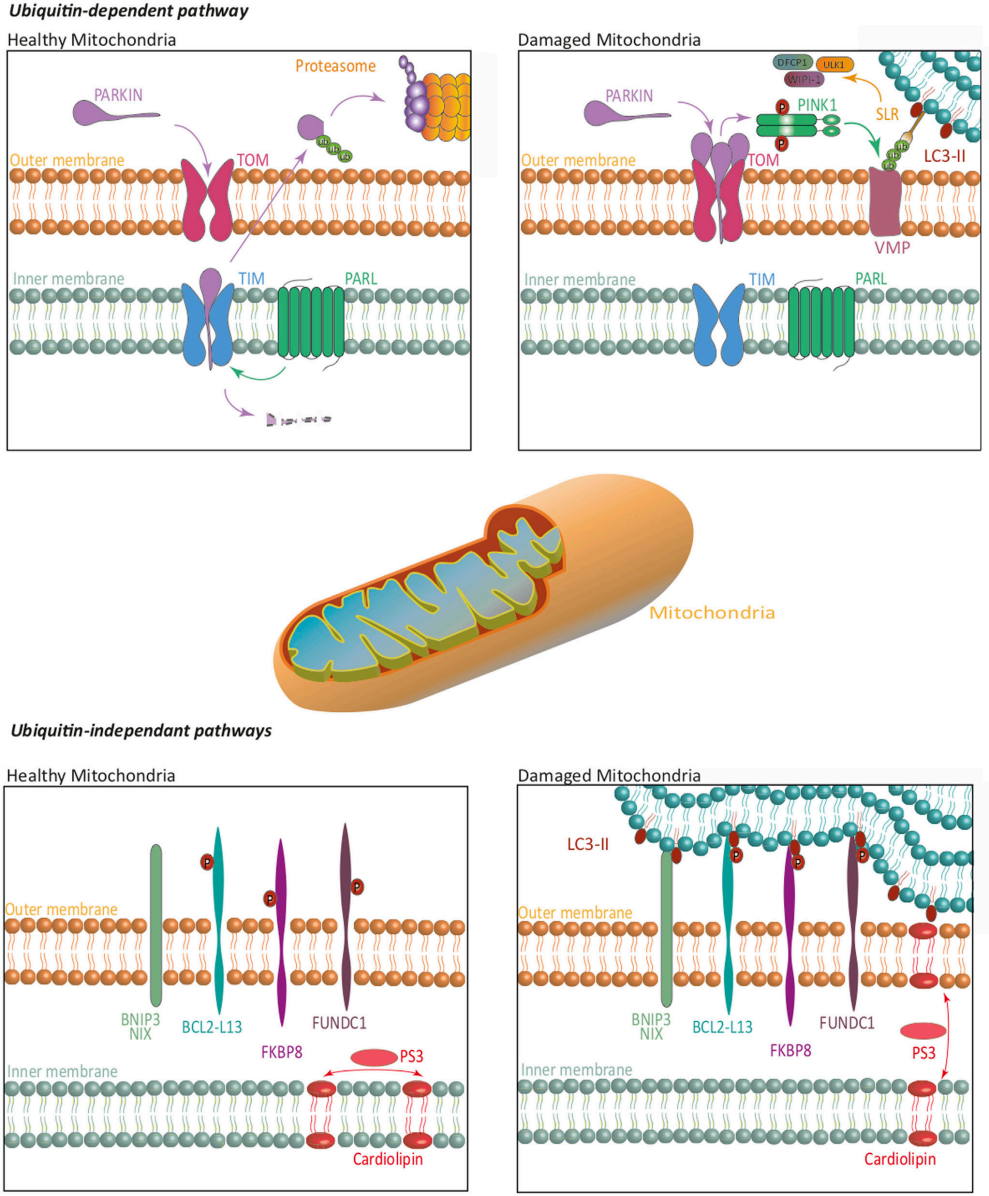
Mitophagy. The ubiquitin-dependent PINK1/Parkin pathway, and ubiquitin-independent pathways regulate mitophagy. On healthy mitochondria, Parkin is constitutively imported and retained on the inner mitochondrial membrane where it is cleaved by PARL, then re-exported for a proteasome-mediated degradation. On damaged mitochondria, Parkin is retained in the outer mitochondrial membrane, where it activates PINK1. Together, they ubiquitinate mitochondrial substrates, leading to their selective recognition by the autophagic machinery. Independently of Parkin, PINK1 induces, *via* NDP52 and optineurin, the recruitment of ULK1, DFCP1, and WIPI-1 to focal spots proximal to mitochondria, to promote mitophagy. In ubiquitin-independent pathways, several receptors are repressed by specific phosphorylations, in healthy mitochondria. Phosphorylations on their LC3-interacting region, lead them to act as degradation signal, and thus recruit the autophagic machinery to insure mitophagy. Some lipids translocation, such as cardiolipin, act in a similar way. The blue lipids layers represent the phagophore membrane. Abbreviations: BCL2-L13, BCL2-like 13; BNIP3, BCL2/adenovirus E1B 19 kDa interacting protein 3; DFCP1, double FYVE-containing protein 1; FKBP8, FK506-binding protein 8; FUNDC1, FUN14 domain containing 1; NIX, Nip3-like *protein* X; PARL, presenilin-associated rhomboid like; PINK1, PTEN-induced putative kinase 1; SLR, sequestosome-like receptor; TIM, translocase of the outer inner; TOM, translocase of the outer membrane; ULK1, Unc-51 like autophagy activating kinase 1; VMP, variety of mitochondrial proteins; WIPI, WD-repeat *protein* interacting with phosphoinositides.

In mammalian cells, mitophagy relies on both ubiquitin-dependent and -independent mechanisms (Figure 2). The first one, which is also the predominant one, is mediated by the PTEN-induced putative kinase 1 (PINK1)/Parkin-mediated pathway (19). PINK1 is imported into the inner membrane of healthy mitochondria in a membrane potential-dependent manner, *via* translocases (translocase of the outer inner and translocase of the outer membrane). In that case, PINK1 is continuously processed by matrix processing peptidases and rhomboid protease presenilin-associated rhomboid like (PARL). This processed form is sensitive to protease-mediated degradation in the cytosol. In case of damaged mitochondria and compromised membrane potential, PINK1 is not imported in the inner membrane but accumulates at the outer membrane, where it is not accessible to PARL-induced degradation. PINK1 is then autophosphorylated and exerts its kinase activity, which allows Parkin recruitment. Parkin belongs to the family of ubiquitin ligases. Parkin recruitment to damaged mitochondria is mediated by PINK1-induced phosphorylation of ubiquitinylated proteins located at the outer membrane of mitochondria. Recruitment of Parkin by phosphorylated ubiquitin leads to the triggering of its ubiquitin ligase activity. Mitochondrial outer membrane proteins then get highly ubiquitinylated and become targets for degradation *via* specific receptors. At least five identified receptors, such as the previously mentioned NBR1 and p62, but also optineurin, TAX binding protein 1, and nuclear domain 10 protein NDP52, recognize these ubiquitin chains. Each of these proteins harbors an LIR motif, suggesting that they can interact with LC3 to recruit the autophagosome membrane, and sequestrate mitochondria for lysosomal degradation. It has also recently been shown that components of the initiation complex could be recruited at the mitochondrial membrane in a PINK1-dependent manner (20).

The first ubiquitin-independent mechanism identified relies on Nip3-like protein X (NIX) or BCL2/adenovirus E1B 19 kDa interacting protein 3 (BNIP3) (18). These molecules play a role in the elimination of mitochondria during erythrocyte development, and in other cells under hypoxia. NIX is localized at the outer membrane of mitochondria and is upregulated during erythrocyte maturation. NIX contains an LIR domain that could allow its interaction with LC3, although other parts of the proteins have been involved. Both NIX and BNIP3 are upregulated during hypoxia and can interact with LC3. Altogether, these pathways direct damaged mitochondria to autophagosomes. NIX and BNIP3 recruitment and activation are regulated by various phosphorylations, although the kinases involved remain to be identified. Bcl2-L13, FUN14 domain containing 1, and FK506-binding protein 8 can also interact with LC3, inducing mitophagy. Interestingly, some mitochondrial lipids might also act as degradation signals by directly interacting with members of the LC3 family (21).

### Autophagy and Proteostasis

**Figure 3:**
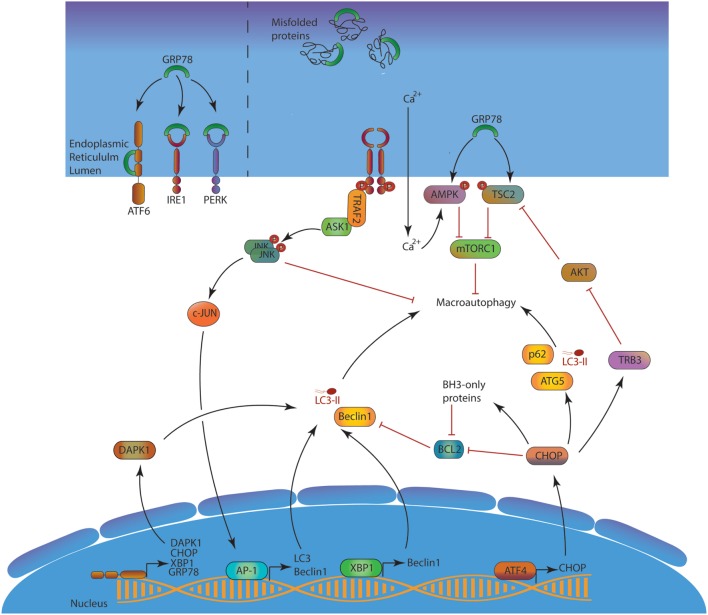
ER stress and macroautophagy induction. In the absence of stress, GRP78 neutralizes the three UPR activation pathways ATF6, IRE1, and PERK. Misfolded proteins accumulation in the lumen creates a competition for GPR78, leading to UPR response activation. Consequently, ATF6 can reach the nucleus, IRE1 splices XBP-1 mRNA to form a functional XBP-1 protein and indirectly activates AP-1, and PERK activates ATF4. ATF6, XBP-1 AP-1, and ATF4 act as transcription factor and induce the expression of several mechanisms to alleviate the ER stress. Among them, autophagy is directly enhanced at two different levels first by the transcription of several *Atgs*, and second at a protein level by inhibiting several autophagy modulators such as BCL-2 or AKT. The blue/purple structures represent, respectively, the nucleus and the ER membranes. Abbreviations: AKT, *protein* kinase B; AMPK, adenosine monophosphate-activated protein kinase; AP-1, activator protein 1; ASK1, apoptosis signal-regulating kinase 1; ATF4/6, activating transcription factor 4/6; BCL-2, B-cell lymphoma 2; CHOP, C/EBP homologous *protein*; DAPK1, death-associated protein kinase 1; GRP78, 78-kDa glucose regulated protein; IRE1, inositol-requiring enzyme 1; JNK, c-Jun N-terminal kinases; mTORC1, mTOR complex 1; PERK, protein kinase RNA-like endoplasmic reticulum kinase; TRAF2, TNF receptor-associated factor 2; TRB3, tribbles pseudokinase 3; TSC2, *tuberous sclerosis complex 2*; *XBP1*, X-box binding protein 1; UPR, unfolded protein response; ATGs, autophagy-related.

Autophagy plays important roles in the equilibrium between protein synthesis and degradation. First, autophagy is induced upon ER stress as a part of the unfolded protein response (UPR, Figure 3) (22). The activation of the protein kinase RNA-like endoplasmic reticulum kinase pathway is known to induce the expression of several ATGs and the macroautophagic process. The autophagic machinery can sequestrate portions of the ER, after recognition of several cargo receptors, namely reticulophagy regulator 1, SEC62, and reticulon 3 (RTN3). These molecules contain LIR domains, allowing linkage to LC3 and sequestration into autophagosomes. This so-called ER-phagy contributes to limit stress and trigger of apoptosis.

Macroautophagy can also selectively degrade proteins outside the ER. Large aggregates are cleared by macroautophagy *via* p62 recruitment, targeting them to autophagosomes. More generally, several reports show a selective degradation of proteins involved in signaling pathways, modulating cell activation and survival.

### Chaperone-Mediated Autophagy

Another well-characterized form of selective autophagy, CMA, consists in the direct translocation of cytosolic content in the lysosome lumen (23). The constitutive chaperone HSPA8/Hsc70, localized at the cytosolic side of lysosomal membrane, can recruit proteins bearing a particular peptide motif [glutamine at one extremity of the motif, one or two positive residues (lysine or arginine), one or two hydrophobic residue, and one negatively charged residue]. This motif is named and KFERQ-like as a reference for the first motif identified in ribonuclease A, targeted to lysosomal degradation. Approximately 40% of proteins in mammals bear such a motif. HSC70 binding to the substrate is modulated by cochaperones like HSC70 interacting protein, heat shock protein 40, and HSC70-HSP90 organizing protein. The HSC70-substrate complex associates with LAMP2a leading to its oligomerization. LAMP2a oligomers are then stabilized by the interaction with HSP90 localized at the luminal side of lysosomes. The substrate is then unfolded and translocates into lysosomes for degradation, with the contribution of luminal HSC70. This selective form of autophagy can be induced during stresses, like nutrient starvation, oxidative stress, DNA damage, and hypoxia (24). Several signaling pathways have also been involved in CMA induction like calcium/nuclear factor of activated T-cells (NFAT) signaling, retinoic acid receptor-α signaling. CMA can also be modulated by mTORC2 complex activity. CMA is involved in protein quality control, generation of amino acids upon starvation, regulation of the cell cycle, and glucose and lipid metabolism (25). It can finally regulate transcription by degrading transcription factors, and signaling intermediates, tuning cell survival and activation.

## The Plasticity of Lymphocytes Implies Metabolic and Structural Adaptations

All lymphocyte subtypes originate from a progenitor named common lymphoid progenitor (CLP), derived from the hematopoietic stem cell (HSC). Although sharing the same origin, these cells display very diverse functions and localizations. Most of them belong to adaptive immunity, while some subsets rely on innate-type detection of danger for activation. Naive lymphocytes of the adaptive immune system are quiescent cells, with low metabolic and transcriptional activity (26, 27). After activation by the antigen receptors, namely B cell receptor (BCR) and T cell receptor (TCR), a dramatic change in both parameters occurs. Naïve cells relying on OXPHOS for survival switch to glycolysis to ensure energy for rapid proliferation and cytokine production. *In vivo*, after an immune response peak, most reactive lymphocytes undergo apoptosis. However, some cells remain as memory cells differentiated from the beginning of the response, or from later effector cells. Memory B and T lymphocyte clones persist for several months or years after immune priming. Most studies point toward a major role for OXPHOS fueled by FAO for long-term survival. Long-lived cells, like memory cells, need to maintain a healthy pool of mitochondria to ensure energy supply, without being affected by damages related to oxidation. During the immune response itself, lymphocytes become effector cells with various functions. Plasma cells differentiated from activated B cells secrete high amounts of antibodies. Helper T cells secrete large quantities of cytokines, and CD8 T cells synthetize lytic granules and become cytotoxic T cells. These changes are possible thanks to high transcriptional activity and capacity for protein synthesis. Cells must then cope with high levels of stress linked to protein synthesis. Finally, nutrient supply and exposure to environmental stress depend on the localization of cells. Naive cells are mainly located in secondary lymphoid organs, while memory cells and innate-like lymphocytes can reside in peripheral tissues, leading them to face different stresses. Consequently, lymphocytes must be very plastic in terms of adaptation to availability of energy source and production, to oxidative stress, and must modulate their synthesis machinery to be able to produce large amounts of proteins. Autophagy is central in these cells, at precise stages of their life, since it regulates ER stress, protein homeostasis, mitochondria function, cell signaling, metabolism, and antigen presentation.

## Macroautophagy in Early Hematopoiesis and Lymphocyte Homeostasis

Studies delineating the role of autophagy in particular cell populations are complicated by the fact that most ATG knockout mouse models are lethal after birth (28). Transfer experiments or conditional deletion models are then required to investigate in detail the role autophagy plays in lymphocyte biology (Table 1). Early studies had evidenced basal macroautophagic activity and its induction upon stimulation in lymphocytes (29, 30).

**Table 1:** Proteins related to autophagy and related functions in lymphocyte populations.

Autophagy-related gene (ATG)	Cell type	Lymphocyte functions	Reference	Pathologies	Reference
*Beclin1*	Development	B and T cell development	(38)		
	T cells	Caspase degradation after T cell receptor (TCR) engagement	(46)	Experimental autoimmune encephalomyelitis	(46)
		T CD4^+^ apoptosis after HIV infection	(30)		

*Atg7*	Development	Hematopoietic stem cell (HSC) survival	(32, 37)		
		Natural killer (NK) cell development and viral clearance	(76)		
		iNK development and mitochondrial homeostasis	(77)		

	T cells	CD4^+^ T cell apoptosis after HIV infection	(30)		
		Survival and metabolic homeostasis of regulatory T (Treg) cells	(73)		
		Regulation of mitochondrial load in mature T cells	(48)		
		Memory T cell maintenance	(52, 57)		
		ER and calcium homeostasis	(45)		
		Degradation of CDKN1B following TCR activation	(65)		
		Energy supply following TCR activation	(60)		

	B cells	Plasmablast differentiation	(85)	Systemic lupus erythematosus (SLE) and lupus-prone mice	(85)
			B-1a B cells survival and metabolic homeostasis	(36, 53, 84)		
			Memory B cell survival	(16, 17)		
		Medullar recirculating memory B cell maintenance	(36, 53)		
	
	iNK cells	Proliferation and survival of developing iNK cells	(78)		

*Atg5*	Development	B cell medullar development	(36)		
		Innate lymphocyte development and survival	(75)		
	
	T cells	CD8^+^ T cell survival	(39, 56)	SLE and lupus-prone mice, multiple sclerosis	(95, 115)
		T cell proliferation following TCR stimulation	(39)		
		Memory CD4^+^ and CD8^+^ T cell maintenance	(52, 56, 57)		
		Mitochondrial load regulation in mature T cells	(25, 50)		
		Survival and metabolic homeostasis of Treg cells	(73)		
		Repression of Th9 differentiation and function	(63)		
	
	B cells	Plasma cell survival	(47, 53)	SLE and lupus-prone mice	(53, 85, 106)
		Mature B cell survival	(53)		
		Toll-like receptor 7 signaling	(106)		
		ER homeostasis and antibody secretion in plasma cell	(47)		
	
	iNK cells	Proliferation and survival of developing iNK cells	(78)		

*Atg3*	T cells	ER and mitochondria homeostasis	(58)		
		Modulation of Nf-κB activation following activation	(64)		
		Repression of Th9 differentiation and function	(63)		

*Atg12*	B cells	Major histocompatibility class II presentation the nuclear viral antigen Epstein–Barr Virus nuclear antigen 1	(87)		

*Atg16L1*	T cells	Survival of intestinal Foxp3^+^ Treg cells	(74)	Crohn’s disease	(74, 92)
*Vps34*	T cells	Reactive oxygen species and mitochondrial load regulation in mature T cells	(51)		
		Peripheral maintenance and function of CD4^+^ Foxp3^+^	(40)		

*WIPI2*	B cells	Mitochondria homeostasis in B cells and germinal center reaction	(54)		

*FOXO1*	Other lymphocytes	NK cell development and viral clearance	(76)		

*FOXO3A*	Development	HSC maintenance	(35)		
*FIP200*	Development	HSC maintenance	(31)		

*LAMP2a*	T cells	Regulation of activation	(79)		

B cells	Endogenous antigen presentation	(89)	Lupus-prone mice	(108)

*Impact on inflammatory pathologies when described are indicated*.

B and T cells are differentiated from HSC localized in the bone marrow, during hematopoiesis. HSC mainly rely on anaerobic metabolism pathways, thus limiting their need for OXPHOS and ROS production. However, their differentiation in CLP requires a metabolism shift to OXPHOS, leading to an increase in energy and ROS production. As a consequence, several studies reported a strong loss of HSC and CLP numbers in the absence of several autophagy genes in these cells (31–33). HSC loss is the result of defective mitochondrial function and DNA damage, leading to an over-activation of caspase-3 and apoptosis (34). These findings are corroborated by the demonstration of the existence of a protective autophagy transcriptional program in HSCs, which lowers their sensitivity to apoptosis (35). These impairments in early hematopoiesis have been reported to induce a blockade after the pro- to pre-B cells transition step and to impair both B and T cell peripheral populations (36, 37). In addition, chimeric mouse models, generated by transfers of *Beclin-1* or *Atg5* KO fetal HSC in *Rag1* KO mice, demonstrated a strong impairment in CLP number and mature B and T cell populations (38, 39). However, in Beclin-1 KO chimeric mice, a decrease of thymocyte populations was described, that was not observed in mice reconstituted with *Atg5* KO cells. This observation suggests that non-autophagic functions of Beclin-1 are crucial during thymic maturation. A role for macroautophagy from thymic development is, however, likely. Later studies demonstrated that macroautophagy impairment leads to an abnormal accumulation of mitochondria in thymocytes that is maintained in peripheral T cells (40). Consequently, ROS are more abundantly produced and are responsible for thymocyte apoptosis, leading to a decrease in thymic and peripheral populations (39). Since Beclin-1 is involved in both mitophagy and apoptosis regulation, its deletion might act at both levels leading to thymocyte death (41).

The study published by Pua et al. gave first insight into the function of autophagy in mature lymphocyte lineage (39). This work showed that lymphocytes from chimeric mice transferred with ATG5-deficient fetal liver hematopoietic progenitors exhibited profound survival and proliferation defects, in both B and T cell populations. This group, and several others, then generated conditional knockout mice with autophagy deficiency after invalidation of several genes: *Atg5, Atg7, Atg3, Beclin-1*, and *Vps34* (36, 42–54). Of note, all studies used promoters highly engaged during early lymphopoiesis: *CD4* or proximal *Lck*, to delete in developing T cells, or *CD19* for B cell lineage-specific deletion. All studies pointed toward a major role for autophagy in naive T cell homeostasis (45, 46, 48, 50, 51). In all models considered, autophagy-deficient thymocytes were reduced in number, but the most striking phenotype is the reduced proportions of T cells in secondary lymphoid organs. As mentioned above, experiments involving fetal liver chimeras demonstrated that B cell differentiation was blocked after the transition between pro to pre-B cell stage, explaining the decrease in B cell number in the periphery (36). These results were discordant with the absence of similar phenotype in *CD19*-cre *Atg5^f/f^* mice, allowing the deletion of this essential autophagy gene from the pro-B cell stage (36, 42–44, 47). Moreover, our work showed that deleting autophagy very early in *Mb1*-cre *Atg5^f/f^mice*, with autophagy invalidation since the pro-B cell stage, only led to a mild decrease in peripheral B cell populations, without developmental abnormalities (55). Interestingly, subsequent studies have shown that some *Atg5* expressing cells still remain in models with ATG deletion driven by *CD19* promoter, which could explain that these mice do not phenocopy chimera models (47). Alternatively, one could argue that hematopoietic cells from chimeric mice harbor a defect in autophagy since the very early steps of hematopoietic differentiation. The work by Simon’s group showed that HSC needed autophagy for survival, and that early differentiation of both lymphoid and myeloid lineages are hampered under ATG7 deficiency (34). It is thus possible that defects in the lymphoid compartments reported in first studies, could be linked to defects accumulated during hematopoiesis and not to intrinsic roles in later developmental stages or in mature peripheral T or B cells.

## Autophagy in Peripheral T Cells

Concomitantly with thymic cellularity reduction observed in first mouse models, several works suggest that autophagy deficiency in T cells is associated with peripheral T cell homeostasis perturbation as well as proliferative defects and increased apoptosis after TCR stimulation. This could be explained by autophagy impact on energy mobilization and organelle homeostasis.

### Macroautophagy in Naïve T Cell Survival

In the periphery, mitophagy might be involved in the maintenance of resting T cell, since naive T cells exhibit a lower mitochondrial load compared with thymocytes isolated from the same mice. Several *Atg* deficiencies lead to an abnormally high mitochondrial load in peripheral T cells (45, 48). Naive T cells produce more ROS, correlated with a higher cell death rate in absence of macroautophagy. In addition, *Atg3*-deficient naive T cells present a more intense rate of apoptosis in long-term culture than wild-type counterparts (45). However, the view of mitophagy as central was challenged, since Beclin-1-deficient T cells do not exhibit any increase in mitochondrial load (46). In this study, Kovacs and colleagues suggest that Beclin-1 might be rather involved in pro-apoptotic protein regulation. However, if mitophagy regulation is truly Beclin-1-independent in T cells remain to be assessed.

Several reports demonstrated a higher sensitivity of CD8^+^ T cells to macroautophagy loss, compared to CD4 T cells (39, 45, 56). Interestingly, CD8 T cells possess a lower mitochondrial load than their CD4 counterpart. This mitochondrial load was reported as increased and associated with higher ROS levels and cell death in *Atg7- or vps34-*deficient CD8 T cells (40, 45). However, the previously quoted papers were based on mouse models presenting an early deletion of *Atgs* (Table 1). In such models, as mentioned before, one could argue that the higher ROS production and cell death observed in mature populations could be for part due to defects accumulated during their developmental phases. After deletion occurring only in mature T cells (56), we confirm CD8^+^ T cells sensibility to ROS linked to defective mitophagy, which is not shared by CD4^+^ T cells. Altogether, these data demonstrate a superior need for mitophagy prior to their activation in CD8^+^ than in CD4^+^ T cells. This requirement for mitophagy could be one of the reasons why CD8^+^ T cells deficient for macroautophagy are globally unable to properly differentiate into memory cells after an immune challenge, as developed later (49, 52, 57).

The importance of proteostasis regulation in peripheral T lymphocytes has first been shown in ATG3- and ATG7-deficient T cells, which harbor an abnormally expanded ER (45, 58). Inducible deletions demonstrated *in vitro* that the progressive accumulation of ER membranes correlated with an increased apoptosis. The ER expansion is accompanied by a defect in calcium storage that could account for part of the apoptotic-prone phenotype and activation defects observed in autophagy-deficient T cells. Thus, autophagy can regulate the amount of ER membranes in T cells.

### Macroautophagy in T Cell Activation

Following T cell activation by TCR engagement, energetic demand dramatically increases. An initial ATP burst is necessary to sustain an increased energetic demand (59). It is mediated by the increase in glucose uptake and glycolysis induction, generating energy and leading to lactate production. 3-methyl-adenine-mediated blockade of macroautophagy induces a decrease of lactate and ATP generation after T cell activation (60). Furthermore, this blockage can be partially restored by the addition of an exogenous source of energy in lymphocytes. Interestingly, after T cell activation, mitochondria and other organelles are spared from autophagic degradation (60). This suggests that T cells use autophagy as a mean to increase energy supply, while maintaining its preexisting production capacity. Moreover, after lymphocyte activation, several signaling pathways are activated. Mitochondrial ROS are themselves responsible for the proper activation of NFAT, notably needed for interleukin-2 (IL-2) production (61). To balance this ROS production, mitogen-activated protein kinase extracellular signal-regulated kinases pathway is responsible for mitophagy induction (62).

The specific degradation of proteins by autophagy also modifies lymphocyte fate. Kovacs and colleagues showed in Beclin-1-deficient T cells that helper T cell subtypes are differentially affected (46). Macroautophagy could also contribute to define helper T cell polarization, through specific protein degradation. IL-9 production by T cells, characteristic of the TH9 profile endowed with antitumoral properties, is under the control of the PU.1 transcription factor. Interestingly, macroautophagy targets PU.1 for degradation, regulating its levels. As a consequence, autophagy-deficient T cells show enhanced TH9-dependent anti-tumor responses (63).

Autophagy can also modulate inflammatory signals in T cells through degradation of signaling intermediates. Indeed, in the absence of autophagy, an accumulation of Bcl-10 mediating NF-kB activation downstream of TCR activation is observed (64). This lowers the threshold for T cell activation. Cell cycle regulators might also be regulated by macroautophagy-mediated degradation. CDKN1B/p27Kip1 can be selectively targeted to lysosomes by macroautophagy (65). After TCR stimulation, macroautophagic degradation of this negative regulator of the cell cycle is increased. Thus, autophagic activity contributes to progression into the cell cycle after T cell stimulation, which could explain proliferative defects described in several ATG-deficient models.

Lipid droplets can also act as modulators of nuclear events, by regulating the nuclear translocation of transcription factor. NFAT-5 is a key transcription factor involved in response to osmotic stress and pro-inflammatory cytokine production. In T-cells, NFAT-5 is activated following TCR stimulation (66, 67). Its knockdown causes a severe decrease of T-cell number (68). Ueno et al. demonstrated that LDs are able to sequester NFAT-5 at their surface thus, blocking its translocation to the nucleus (69). The expending importance of LDs in the immune system and proteins/pathways regulating their formation and degradation, make them interesting targets to modulate the immune system activity. It is elegant to think that *via* lipophagy, macroautophagy could directly modulate the activity of transcription factor such as NFAT-5. However, no proof of this concept has yet been reported.

The role of autophagy early after T cell activation has been controverted after important findings from Ahmed’s team (52). Previously described studies had postulated that TCR engagement led to macroautophagy activation to support energetic demand. By rigorously assessing autophagic flux *in vivo* during the dynamics of CD8 T cell responses, Xu and colleagues found instead that the accumulation of autophagic markers previously observed in T cells early after activation is mainly due to autophagic blockade at final stages. This assumption makes sense, as naive cells possess, before activation, few energetic stores. Extensive degradation of these stocks could then be detrimental to cell survival. Thus, T cells activated trough the TCR would limit autophagic degradation while requiring exogenous energy source, quickly mobilized through glycolysis. Nevertheless, other reports focused on CD4^+^ T cells show that autophagy can be induced in particular settings. Botbol and colleagues showed a crucial role for gamma-chain-associated cytokine receptor in macroautophagy induction, through a post-transcriptional mechanism (70). Cytokines like IL-2, IL-4, IL-7, and IL-15 are potent inducers of helper T cell activation. It is possible that CD4^+^ and CD8^+^ T cell differ in their autophagy requirement and sensitivity to cytokines stimulation at first steps of activation. It is also possible that CD8^+^ T cells sensitivity to cytokine-induced autophagy is visible later during immune response. Interestingly, IL-2 is not mandatory for primary CD8 responses but essential for the generation of memory cells. We can thus speculate that autophagy activation in response to IL-2 occurs several days after initial CD8^+^ T cell activation, at the effector phase, supporting memory T cell generation.

In accordance with a non-essential role for autophagy in early T cell activation, several works using the previously cited models suggest that deficiency of autophagy in mature T lymphocytes poorly impact their function at short term (42, 43, 52, 57). Among them, our work studying dLck-cre *Atg5^f/f^* mice showed that autophagy was dispensable for short-term CD4^+^ T cell activation.

### Macroautophagy in Memory T Cells

The most recent studies suggest a preferential role for autophagy in certain lymphocyte subtypes. It is now clear that autophagy allows the persistence of both CD4 and CD8 memory T cells (42, 43, 52, 57). Using transfer mouse models, or granzyme-cre *Atg7^f/f^* mice allowing the suppression of autophagy only in effector CD8^+^ T cells, three groups reported a major role for autophagy in memory T cell survival.

Autophagy requirement for memory maintenance could be explained by the switch that T cells operate toward OXPHOS linked to high FAO activity, at the transition from effector to memory phase (71). Metabolomic and transcriptomic studies revealed that autophagy-deficient T cells exhibit aberrant lipid content profile as well as deregulated enzymatic pathways related to lipid metabolism (52). The carnitine shuttle and di-unsaturated fatty acid β-oxidation are impaired in autophagy-deficient T cells. The authors suggest that defects in metabolite generation under macroautophagy deficiency selectively impair memory T cell survival. Autophagy could support CD8^+^ T cell lipid metabolism trough lipophagy, as recently shown for neutrophil development (72). This could also be true for memory CD4^+^ T cells. We have described that autophagy-deficient CD4 T^+^ cell accumulate neutral lipids, which could be linked to defective lipophagy (56).

### Macroautophagy in Regulatory T (Treg) Cells and Innate Lymphoid Cells

Other lymphocyte subsets might rely on autophagy for mobilization of lipids. Treg-specific autophagy invalidation leads to glycolysis over-activation, compromising their functions (73). Autophagy might thus contribute to metabolic balance in these cells. Other reports showed that Treg cells (73, 74), innate lymphoid cells that include natural killer cells (75, 76), NKT cells (40, 77, 78) strongly rely on autophagy for survival and differentiation.

### CMA in Peripheral T Cells

Chaperone-mediated autophagy has also been shown to participate in T lymphocyte homeostasis. Macian’s group reported that T cells deficient for LAMP2a exhibited a reduced responsiveness upon stimulation, limiting the magnitude of T cell-related immune responses (79). CMA can also modulate the extent of T cell activation, through selective degradation of the negative regulators ITCH and regulator of calcineurin (RCAN) (79). In that case, CMA deficiency in T cells compromises activation after stimulation.

## Autophagy in Peripheral B Cells

### Macroautophagy in Peripheral B Cell Survival and Activation

First studies were discordant regarding the role of autophagy in the maintenance and activation of peripheral B cells. Some studies showed that autophagy was not required for proper B cell survival or activation (36, 80). Another study showed, however, that BCR associated with costimulation was able to activate autophagy although the physiological relevance of this phenomenon was not clear (81). Some studies suggest that primary immune responses are poorly impacted by autophagy deletion (42), while decrease in T cell-independent and -dependent responses have been described in others (44). A defect in plasma cell generation was also reported in the latter study after infections in the absence of autophagy in B cells. These discrepancies could be due to the model used (deletions of ATG5 or ATG7) and the immunization protocol (infection, model antigens). For example, the use of Mb1- and CD21-cre mediated ATG5 deletion, leads to a small decrease of peripheral B cell populations in contrast to previous models (53).

Several arguments point toward a role for autophagy in B cell function and survival restricted to some activation stages. Recently, Batista’s group observed that germinal center (GC) B cells were the most active in processing autophagy. They reported alterations of GC when WD repeat domain, phosphoinositide-interacting protein 2 (WIPI2) was deleted in B cells. Interestingly, deficiency in ATG16L1 did not lead to such a defect. WIPI2 is needed for terminal B cell differentiation and negatively regulates non-canonical forms of autophagy (82). Other non-canonical autophagic pathways such as LC3-assisted phagocytosis (LAP) are described, with components common with canonical autophagy, some unshared with autophagy, and specific ones like Rubicon (83). Similar pathways could also be activated in GC B cells. In the latter work, Martinez-Martin and co-authors also found more antibody-secreting cells under WIPI2 deficiency. The authors postulate that WIPI2 is an important regulator of mitophagy in B cells, regulating GC organization and the outcome of B cells. Mitophagy in B cells could then require a particular machinery. Additional studies are required to elucidate the contribution of different ATGs and their partners in B cell mitophagy.

Indeed, little is known concerning the role of macroautophagy in mitochondrial homeostasis in B lymphocytes. We and others did not detect any impact of core autophagy gene deletion (*Atg5* and *Atg16l1*) in mitochondrial content in naive B cells (53, 54).

As for mitophagy, only few is known about lipophagy in B-2 B cells. In a recent report, B-1a B cells were described as relying on lipophagy for their metabolic homeostasis and their self-renewal (84). This could explain the preferential decrease in this cell population among B cells, under ATG5 deficiency (36, 44, 47, 53). Mature recirculating B cells in the bone marrow (Fraction F according to Hardy’s nomenclature) also seem dependent on autophagy for their maintenance, although the mechanisms explaining the preferential role for autophagy in that population remains to be defined (36, 53). NFAT-5 activity modulation by lipophagy could also play a role in B cell activation, in particular in response to B cell activation factor, as NFAT-5 KO leads to defects in immunoglobulins G production (68).

### Macroautophagy in Memory B Cell and Plasma Cell Survival

In contrast to the discussed role of autophagy in naïve B cells, the high dependence on autophagic activity of memory B cells (42, 43) and plasma cells (44, 47, 53) is well described.

Mice with ATG7-deficient B cells infected by influenza virus are able to mount a normal primary immune response (42). They, however, fail to generate a protective secondary response upon a second viral challenge leading to increased mortality rates. Another article from the same group showed later that autophagy is involved in the maintenance of memory cells and not on their generation in that context (43). Chen et al. (42) showed that autophagy in memory B cells limits mitochondrial ROS production and toxicity of peroxidized lipids. It is also possible that mobilization of lipids through lipophagy might be required for the survival of both memory B and T cells.

Plasma cells, another late differentiation stage in the B cell lineage is also dependent on autophagy. Indeed, plasma cells are characterized by a large ER compartment compared with their B cell precursor (47). As previously mentioned, the high secretory activity of these cells exposes them to elevated levels of ER stress. These cells thus highly express several effectors of the UPR, some of which are known to induce macroautophagy. Several reports highlighted the particular role played by autophagy in the maintenance of plasma cell compartment (44, 47, 53, 85). Among them, Pengo and collaborators, showed an ER expansion in autophagy-deficient plasma cells. Although leading to an increased IgM secretion at short term, this expansion might lead to an apoptotic-prone phenotype, consequently to an uncompensated UPR response. It is also possible that mitophagy contributes to plasma cell survival. We indeed observed a slight increase in mitochondrial load and decreased membrane potential in plasmablasts differentiated after lipopolysaccharide stimulation (53). It is thus possible that plasma cells need macroautophagy to optimize their mitochondrial pool. Whereas several authors agree on the role of macroautophagy in plasma cell maintenance, if autophagy impacts early events, leading to plasma cell fate still remains unclear. Conway and collaborators report early defects in plasma cell markers after immunization (44). Batista’s group shows that the balance between canonical and non-canonical autophagy in the GC affects B cell terminal differentiation (54). Further studies are needed to fully understand the role of autophagy in early plasma cell differentiation.

### Autophagy and Antigen Presentation by B Cells

B cells are antigen-presenting cells (APCs) expressing high basal levels of major histocompatibility class II (MHC-II) molecules. They are poorly competent at activating naive T cells. They need, however, to present antigens to primed CD4^+^ T cells in so-called T cell-dependent responses, for terminal differentiation into memory or plasma cells, and generation of high affinity antibodies. One major source of presented antigens comes from the ones internalized after recognition *via* the BCR. Presentation to cognate T cells allows the final maturation of B cells in GC. Early studies had shown that B cell lines also presented significant amounts of peptides coming from intracellular sources, on MHC-II molecules (86). Interestingly, B cell starvation led to increased proportions of such antigens, suggesting a contribution of macroautophagy in that process. Munz’s team provided a functional insight in the presentation of endogenous antigens, by demonstrating that the nuclear antigen Epstein–Barr Virus nuclear antigen 1 is efficiently processed on MHC-II for presentation to T cells *via* macroautophagy (87). The relevance of basal or induced presentation of other viral antigens for the induction of immune responses by B cells remains to be investigated. It has also been shown on other APC, like dendritic cells, that autophagy is integral for the initiation of immune response during herpes simplex virus infection (88). CMA has also been shown to contribute to the presentation of intracellular antigens. Blum’s team showed that peptides derived from GAD enzyme were translocated *via* CMA to lysosomes and MHC-II compartments for processing and presentation (89). However, the role played by CMA in B cells at the onset of humoral response, remains to be established.

Aside the presentation of endogenous antigens, ATGs have been proposed to play a role in the processing of antigens internalized after recognition by the BCR. Chaturvedi and colleagues described that vesicles with an autophagosomal morphology colocalize with the internalized BCR (90). They postulated that macroautophagy was necessary for trafficking of BCR-containing endosomes toward toll-like receptor 9 (TLR9) positive compartments. A few years later, Unanue’s group showed that LC3 colocalize with the internalized BCR (91). They also found that 3-MA treatment in B cells impaired the citrullination of antigens. They hypothesized that macroautophagy allows the trafficking of internalized BCR to protein arginine deaminase-containing compartments, which mediate citrullination. They also noticed a small decrease in non-citrullinated antigen presentation. It is thus possible that macroautophagy contributes to BCR trafficking for signaling or antigen processing. It could, however, appear paradoxical that most mouse models with B-cell-specific autophagy deficiency show weak impairment of primary humoral responses after T cell-dependent antigen challenges or infection. Indeed, presentation of antigens acquired through BCR internalization is needed to require help by T cells to enter GC. Martinez-Martin and colleagues show a balance between canonical form of autophagy on WIPI2 and non-canonical autophagy (54). The latter form could involve LC3 recruitment at BCR sites of endocytosis. The authors propose that only part of the autophagy machinery would contribute to BCR trafficking after endocytosis for optimal antigen processing. This process would be reminiscent of processes like LAP. Alternatively, our recent experiments with ATG5-deficient B cells show that a Beclin-1-Vps34-dependent pathway is integral to centrosome relocalization after BCR engagement (Arbogast et al., in press). This B cell polarization is needed for optimal acquisition of immobilized antigens *in vivo* and *in vitro*. Thus, macroautophagy, or part of its machinery, might tune BCR trafficking to optimize antigen processing under particular circumstances.

## Lymphocyte Autophagy in Chronic Inflammatory Diseases

First indications about an involvement of macroautophagy in inflammatory diseases came from genome-wide association studies underlining a link between proteins of the autophagy machinery and Crohn’s disease. In this chronic gut autoinflammatory disorder, variant forms of ATG16L1 are among the highest susceptibility marker. Deficiencies in this core ATG protein could lead to impaired Paneth cells secretions of antibacterial peptides, hyperactivation of the inflammasome, and impaired antigen presentation by APCs as reviewed in Ref. (92). The T300A variant, found in a subgroup of patients, does not impair all macroautophagy processes, but leads to hyporesponsiveness to NOD2 stimulation, and decrease in the capacity to degrade invasive bacteria through macroautophagy. This variant can also increase ATG16L1 degradation by caspases. Finally, it could lead to defects not linked to autophagy deregulation. Regarding T cells, a recent study by Maloy’s team showed that T cell-specific ATG16L1 deficiency led to an aberrant type 2 inflammatory response toward bacterial antigens (74). Moreover, specific autophagy ablation in Treg cells leads to metabolic defects, which could explain the observed hyper-inflammation. Later studies identified polymorphisms in other genes linked to macroautophagy such as IRGM and ULK1, as recently reviewed in Ref. (93). Their precise impact on T cell biology in the intestine remains to be investigated.

The search for a role of autophagy during lupus has gained in interest in recent years. Treatments like rapamycin or hydroxychloroquine (HCQ), modulating lupus activity, showed beneficial effects. Moreover, polymorphisms in *IRGM* and *ATG5* polymorphisms, and variations in *PRDM1-ATG5* intergenic region have been associated with systemic lupus erythematosus (SLE) (94, 95). SLE is characterized by the activation of auto reactive lymphocytes, which induce the production of autoantibodies mainly directed against nuclear antigens. These antibodies induce local inflammation damaging several tissues, like blood vessels, skin, kidney, and central nervous system (CNS). The functional consequences of these polymorphisms need to be addressed, as they are not located in coding regions. They could translate the existence of other polymorphisms in particular alleles in subgroups of patients, with detrimental effects in terms of susceptibility to SLE. One study showed that *ATG5-PRDM1* allelic variant was associated with increased *ATG5* expression (95). An earlier work showed that addition of antibodies purified from serum of SLE patients to cell lines lead to an increase in autophagic markers (96). Recent important findings endowed non-canonical forms of autophagy with a role in cell clearance by phagocytes (97), proposed to be defective during SLE.

First studies investigating in detail the autophagic activity of lymphocytes in SLE patients reported an increase in the autophagic marker LC3-II (98–100). These studies diverge in their explanation for the accumulation of autophagic markers. Alessandri and colleagues conclude about a blockade of the autophagic flux, which could sensitize cells to apoptosis, a common feature of lupus T cells. They also reported in a later study the accumulation of α-synuclein linked to autophagy impairment in lupus T cells (101). Another work showed that impaired autophagy in lupus T cells leads to an apoptosis-prone phenotype due to increased ER stress (102). On the contrary, other studies (85, 98), and a more recent one (103), hypothesized that autophagy could contribute to the survival of activated T cells. Indeed, they found that the flux is not totally blocked in lupus T cells. It is clear, however, that aberrant macroautophagy occurs in lupus T cells, leading to an imbalance between the generation of autophagosomes and their degradation. Alternatively, macroautophagy could be impaired in some T cell subtypes and not in others. Kato and Perl recently found that autophagy was suppressed in Treg cells from SLE patients, in response to mTOR activation triggered by IL-21 signaling (104). A previous study had shown that suppression of DEF-6 and SWAP200 in lupus-prone mice allow an increased expression in interferon-regulated factor 4, leading to an augmentation of the Treg compartment and amelioration of their function, mitigating the disease (105). This improved function was associated with an increased autophagy gene expression. To reconcile these different views, we could argue that it is possible that in some populations, like memory cells, macroautophagy contributes to survival and chronic activation. In some others like naive cells, or Treg cells, the continuous generation of autophagosomes is not balanced by their degradation and leads to cell death.

In B cell lineage from SLE patients and lupus-prone mice, a higher autophagic activity was observed in precursors and naive B cells (85). Macroautophagy was shown to favor survival of plasmablasts and plasma cells, contributing to the production of autoantibodies (53, 85). It was further shown that autophagy in B cells was integral to lupus development in a TLR7 overexpression mouse model (106). The authors postulate that macroautophagy might allow the translocation of RNA-containing antigens to TLR7 positive compartments, in a similar way to what was observed for TLR9 (90). The contribution of macroautophagy to memory B cell survival in chronic inflammation, although plausible, remains to be assessed during lupus. Macroautophagy could also contribute to autoantigens presentation by B cells during SLE. However, to date, no study precisely addressed this question. CMA has also been proposed to contribute to autoantigen presentation. The phosphopeptide P140, which efficacy was recently tested in phase 3 clinical trial for SLE, inhibits both CMA and macroautophagy in this context (107, 108). The authors of this work propose that limiting these autophagy pathways could limit autoantigen presentation. They indeed report a decrease in MHC-II molecules expression by B cells (103, 107, 109). Further studies are needed to confirm efficacy shown in phase IIb and to precisely define which types of antigens are concerned.

The role of autophagy has been addressed in several cell types in another systemic autoimmune disease: rheumatoid arthritis (RA). Macroautophagy has been involved in the deregulation of fibroblast, chondrocyte, macrophage, and osteoclast homeostasis (110). Most studies point toward a detrimental role for macroautophagy protecting, for example, inflammatory fibroblasts from ER stress-induced cell death and favoring osteoclastogenesis. In addition to previously discussed potential roles in memory lymphocyte survival, these variations might affect antigen presentation. Indeed, one hallmark of RA is the development of antibodies directed against citrullinated epitopes. As mentioned before, presentation of citrullinated antigens by B cells need the contribution of macroautophagy (91). The physiopathological relevance of this phenomenon is not known. Other studies reported that macroautophagy was impaired in T cells from RA patients (111). Macroautophagy inhibition was linked to the insufficient induction of phosphofructokinase, an enzyme favoring glycolysis. Thus, RA T cells are prone to apoptosis and senescence due to insufficient energy supply through glycolysis and macroautophagy mobilization. A recent study seems to contradict the previous findings, showing that autophagy is increased in CD4^+^ T cells isolated from RA patients and mouse models (112). The authors argue that in their settings, CD4^+^ T cells are assessed for autophagic activity directly after isolation, whereas in previous study cells were cultured for 48 h before macroautophagy assessment. Moreover, increased autophagic activity is found in total T cells in contrast to the study by Yang and colleagues who worked with naive cells. It is thus possible that, as for lupus, deregulation of macroautophagic activity is more linked to certain lymphocytes subtypes like memory cells.

Macroautophagy involvement has also been proposed in an organ-specific autoimmune pathology. Multiple sclerosis (MS) is characterized by chronic inflammation and demyelination in the CNS. An initial inflammation leads to leukocyte infiltration into the CNS. There, residents APCs present myelin-derived antigens, probably originate from dying oligodendrocytes, leading to the priming of T cells. Autoreactive T cells then contribute to the pathology by significantly contributing to inflammation and inducing further oligodendrocytes death (113). In MS context, autophagy roles have been notably assessed in neurons and APCs, however, only few is currently known in lymphocyte populations. However, an elevated autophagic flux has been reported in these autoreactive T cells, both in patients and in the mouse model of experimental autoimmune encephalomyelitis (EAE) (114). It might play a non-negligible role in the pathology onset, since mice deficient for *Beclin-1* in T cells are resistant to EAE development (46). Such a resistance might be link to a decrease in CD8 T cell number. Furthermore, Th1 cells that can contribute to the pathology are more susceptible to Beclin-1 loss than other subtypes. It might further limit CD8 T cells priming. The limited impact of autophagy invalidation on TH17 could appear surprising, as these cells are major actor in EAE pathology. It is thus possible that the global decrease in T cell response under Beclin 1 deficiency is sufficient to limit the disease. A recent report demonstrated that *ATG5* mRNA is increased in CD4^+^ T cells isolated from MS patients (115). Interestingly, they demonstrated that this increase is independent on autophagic activity, but rather correlated with other inflammatory cytokine levels, such as tumor necrosis factor-alpha. Taken altogether, these results suggest a non-canonical role for autophagic machinery in the onset of MS. However, further studies are necessary to fully understand how autophagy in T cells might contribute to MS severity. As B cells are proposed as important actors in MS (116), assessing autophagic activity in these lymphocytes would also be of interest.

## Modulating Lymphocyte Autophagy as a Therapeutic Strategy for Inflammatory Diseases

In the last decade, several pharmacological agents were reported as modulators of the autophagic activity. However, a large majority of these compounds were elaborated and tested *in vitro*, and only few succeeded to pass or are currently in clinical trials. In this last part, we will discuss those that are currently used, or in trial, to either activates or inhibits autophagy in the previously mentioned pathologies. Even if not proven, several actions of described compounds could be linked to autophagy impairment.

Inhibiting autophagy could appear beneficial in some settings. Chloroquine (CQ) and HCQ are two synthetic agents blocking autophagy used notably as anti-malaria treatment. CQ and HCQ, as lysosomotropic agent, raise the intralysosomal pH, thus impairing autophagosome degradation. Since years CQ and HCQ are used during SLE and RA. Among their many actions, they impair MHC-II-mediated presentation, thus diminishing antigen presentation to CD4 T cells. This impairment seems to favor cross-presentation, and thus the priming of naive CD8 T cells (117). However, in humans CQ usage seems to induce a systemic reduction of both CD4 and CD8 T cells (118). In addition, CQ inhibits endosomal TLR responses, which are particularly implied in SLE and RA, notably in DNA and RNA recognition (119) CQ was also used during early studies about mechanism of lipid degradation in lysosomes, which were impaired during treatment (120). A recent report demonstrated that CQ induces apoptosis *via* ER stress (121). Even if all the effects induced by CQ/HCQ treatment are not currently known, they might act on several levers to modulate lymphocyte overreactions. First, by inhibiting T cell activation, and second potently by inducing several homeostatic challenges that might be damageable for B and T memory cells and plasma cells. However, due to its severe unwanted effects, CQ and HCQ are not fully satisfying for after long-term treatment for some patients. Thus, researches for novel autophagy inhibitors are still needed. Sphingosine-1-phosphate and its analog, fingolimod (FTY720) have been demonstrated to be mTOR/p70S6K pathway activators, thus indirect inhibitors of macroautophagy. Used in MS, it impairs CD8 T cells functions (122). However, its exact mechanism of action on these cells remains currently unknown.

Activating autophagy could also appear as a suitable strategy in several settings. Autophagy activators might be discriminated on two main classes. Some are activating autophagy by an inhibition of mTOR, and some others, which are mTOR-independent. Among the mTOR inhibitors, several drugs are currently envisaged for therapy, such as rapamycin, resveratrol, metformin, chlorpromazine, lithium, minocycline, and valproic acid. Even if these drugs do have potential as autophagy inducers, they also possess plenty of side effects. Rapamycin and Rapalogs are notably identified as immuno-suppressors (123). Therefore, even if their usage on mouse models has been rather conclusive, their relevance in human health remains to be fully investigated. mTOR-independent autophagy activators act on several other levers, such as Clonidine, a K^+^ ATP channel opener or Verapamil a Calcium channel antagonist. Their mechanisms of action on autophagy is then probably more relying on AMPK–ULK pathway. However, these indirect actions on autophagy might also induce complications, notably in tissues highly relying on ATP and calcium such as the nervous and cardiac systems. Comparably to autophagy inhibitors, the lack of specificity and the spectrum of side effects, complicate their potential *in vivo* usage. Autophagy inducers might enhance notably memory cells functions and long-term survival which could be detrimental to disease progression. It could in contrast favor Treg cell survival and restore naïve cell homeostasis which would limit inflammation.

## Conclusion

Important progresses have been made in our understanding of autophagy involvement in distinct lymphocyte subtypes. Several fundamental questions remain unanswered. First, why naïve CD8 T cells seem to be more dependent on autophagy than CD4 T cells? Moreover, is autophagy really induced at the beginning of T cell activation or rather inhibited as alternatively proposed in Ref. (52)? What are the signaling pathways downstream of the TCR that are responsible for autophagy modulation and how can they collaborate with cytokine-induced signaling? On the B cell side, it is still not known by which mean autophagy mediates memory B cell survival. It has been proposed that autophagy is responsible for survival of plasma cells sensitive to ER stress. Is it the only contribution of autophagy? Does mitophagy participate in plasma cell survival, or in metabolism regulation? In these cells, a very complex picture is emerging regarding autophagy regulation after activation, involving both canonical and non-canonical mechanisms. Their respective contributions to cell survival, receptor trafficking, antigen presentation remains to be assessed and the signaling pathways involved to be determined. Finally, the contribution of ATG proteins in intracellular trafficking, synapse formation, especially in cytotoxic mechanisms could be investigated. In any case, the first discoveries unraveling a major role for autophagy in secondary responses could help to optimize vaccination strategies.

Regarding autoimmune pathologies, it is complicated to draw a general picture of lymphocyte autophagy deregulation during autoimmunity. It can be over-activated or impaired according to the context and the cell subtype studied. Inhibiting autophagy to deplete memory autoreactive cells, or antibody producing cells like plasma cells is a seducing idea. Indeed, such approaches are envisaged for plasma cell depletion with bortezomib, leading to increased ER stress in this sensitive population. This kind of strategy needs to take into account a very narrow therapeutic window to avoid toxicity toward other cells. It appears also crucial to understand if current therapies modulating autophagy like HCQ exert their effect at least in part through autophagy regulation. Monitoring of autophagic activity under treatment, in different lymphocyte subtypes would be important to define the contribution of autophagy modulation. One must keep in mind that inhibiting autophagy can have deleterious effects in certain pathologies. It could increase the apoptotic phenotype of autoimmune cells like in SLE and contribute to the generation of new cell debris fueling inflammation. Moreover, broad inhibition of autophagy mechanisms could impact LAP and thus efferocytosis, which would increase inflammation. LAP relevance in SLE patients remains to be assessed but if its contribution is confirmed, developing agents targeting specific autophagy effectors, unnecessary for LAP could be an option. Finally, some autoimmune pathologies like systemic sclerosis are linked to polymorphisms in *ATG* genes. No information is available to date on autophagic activity in lymphocytes. It appears very important to define new strategies for this pathology as treatment options are scarce. These studies will be fundamental to envisage the use of more specific autophagy modulators for each autoimmune pathology, where autophagy deregulation in lymphocytes is a described feature. Targeting autophagy in lymphocytes is a unique occasion to target precisely memory or effector lymphocytes, to limit chronic inflammation.

## Author Contributions

FG and FA wrote the article. FA prepared the illustrations.

## Conflict of Interest Statement

The authors declare that the research was conducted in the absence of any commercial or financial relationships that could be construed as a potential conflict of interest.
