# Corrigendum: Lymphocyte Autophagy in Homeostasis, Activation, and Inflammatory Diseases

**DOI:** 10.3389/fimmu.2018.02627

**Published:** 2018-11-16

**Authors:** Florent Arbogast, Frédéric Gros

**Affiliations:** ^1^CNRS UPR3572, Immunology, Immunopathology and Therapeutic Chemistry/Laboratory of Excellence MEDALIS, Institut de Biologie Moléculaire et Cellulaire, Strasbourg, France; ^2^University of Strasbourg, Strasbourg, France

**Keywords:** autophagy, mitophagy, metabolism, unfolded protein response, autoimmunity, lymphocytes

In the original article, two clarifications about cited references are necessary.

First, the sentence “As a consequence, autophagy-deficient T cells show impaired TH9 differentiation and antitumor responses (63)” should be “As a consequence, autophagy-deficient T cells show enhanced TH9-dependent anti-tumor responses (63)”. A correction has been made to the section *Autophagy in Peripheral T Cells, Macroautophagy in T Cell Activation*, paragraph 2.

Moreover, even if mechanisms are not totally understood, Chen et al. indeed found experimental evidence in [42], for a role played by autophagy in limiting lipid peroxidation toxicity induced by reactive oxygen species. The sentence “To date, no mechanism linking autophagy and memory B cell survival has been proposed. It is possible that mitophagy and mobilization of lipids through lipophagy might be important, as for T cells” has been corrected to “Chen et al. (42) showed that autophagy in memory B cells limits mitochondrial ROS production and toxicity of peroxidized lipids. It is also possible that mobilization of lipids through lipophagy might be required for the survival of both memory B and T cells”. A correction has been made to the section *Autophagy in peripheral B Cells, Macroautophagy in Memory B Cell and Plasma Cell Survival*, paragraph 2.

The authors apologize for these errors and state that they do not change the scientific conclusions of the article in any way. The original article has been updated.

## Conflict of interest statement

The authors declare that the research was conducted in the absence of any commercial or financial relationships that could be construed as a potential conflict of interest.
